# AM1241 alleviates MPTP-induced Parkinson's disease and promotes the regeneration of DA neurons in PD mice

**DOI:** 10.18632/oncotarget.18871

**Published:** 2017-06-29

**Authors:** Jun Shi, Qiong Cai, Jingxing Zhang, Xiaolie He, Yigang Liu, Rongrong Zhu, Lingjing Jin

**Affiliations:** ^1^ Department of Neurology, Tongji Hospital, Tongji University School of Medicine, Tongji University, Shanghai 200065, China; ^2^ College of Environmental Science and Engineering, Tongji University, Shanghai 200092, China; ^3^ School of Life Science and Technology, Tongji University, Shanghai 200092, China

**Keywords:** AM1241, PD, MPTP, PI3K/AKT, neurogenesis

## Abstract

The main pathological feature of Parkinson's disease (PD) is the loss of dopaminergic neurons in the substantia nigra. In this study, we investigated the role of cannabinoid receptor 2 (CB2R) agonist AM1241 on 1-methyl-4-phenyl-1,2,3,6-tetrahydropyridine (MPTP)-induced neurotoxicity in a mouse model of PD. Upon treatment with AM1241, the decreased CB2R level in the PD mouse brain was reversed and the behavior score markedly elevated, accompanied with a dose-dependent increase of dopamine and serotonin. In addition, western blot assay and immunostaining results suggested that AM1241 significantly activated PI3K/Akt/MEK phosphorylation and increased the expression of Parkin and PINK1, both in the substantia nigra and hippocampus. The mRNA expression analysis further demonstrated that AM1241 increased expression of the CB2R and activated Parkin/PINK1 signaling pathways. Furthermore, the increased number of TH-positive cells in the substantia nigra indicated that AM1241 regenerated DA neurons in PD mice, and could therefore be a potential candidate for PD treatment. The clear co-localization of CB2R and DA neurons suggested that AM1241 targeted CB2R, thus also identifying a novel target for PD treatment. In conclusion, the selective CB2 agonist AM1241 has a significant therapeutic effect on PD mice and resulted in regeneration of DA neurons following MPTP-induced neurotoxicity. The possible mechanisms underlying the neurogenesis effect of AM1241 might be the induction of CB2R expression and an increase in phosphorylation of the PI3K/AKT signaling pathway.

## INTRODUCTION

Parkinson's disease (PD) is a neurodegenerative movement disorder that is characterized primarily by a massive loss and degeneration of dopaminergic (DA) neurons in the substantia nigra compacta (SNc) and a significant reduction of striatal dopamine [1]. At present, the main drug treatment for PD is the replacement of dopamine with levodopa (L-dopa); however, long-term use of L-dopa is often associated with disabling fluctuations and dyskinesias, negating its beneficial effects [2]. Thus, to avoid the motor complications arising with use of L-dopa, ongoing research pursues the development of new non-dopaminergic drugs that are able to arrest and even reverse the degeneration of dopaminergic neurons without causing dyskinesia.

Endocannabinoids are lipid signals that exert most of their actions via activation of specific G-protein-coupled receptors—type 1 and type 2 cannabinoid receptors (CB1R and CB2R) [3–4]. Previous studies have identified cannabinoids as an interesting class of drugs with neuroprotective properties against excitotoxicity and oxidative stress neuroinflammation, which are also associated with PD [5]. In recent years, CB2R has been identified in astrocytes, oligodendrocytes, quiescent and perivascular microglia, neural progenitors, and even in a few subpopulations of neurons. Furthermore, the CB2R has been found in the substantia nigra of patients with PD, albeit at a significantly lower level than non-PD controls [6]. CB2R has been recently reported to modulate brain dopamine-related behaviors by JWH133 [7]. Moreover, *in vivo* studies have shown that pharmacological activation of CB2Rs by JWH015 can reduce microglial activation, neurodegeneration, and the emergence of functional deficits in mouse models of PD [8].

Among the agonists of the CB2R, AM1241 is typical and specific, and has been reported to relieve migraine [9], stroke [10], and neuropathic pain [11]. Furthermore, one recent study showed that AM1241 could functionally enhance neurogenesis in the hippocampus of GFAP/GP120 transgenic mice [12]. However, few studies have focused on the therapeutic effect of AM1241 in PD and the regeneration of injured DA neurons, and the underlying mechanisms remain unexplored.

Therefore, in this study, we investigated the therapeutic effect of AM1241 on 1-methyl-4-phenyl-1,2,3,6-tetrahydropyridine (MPTP)-induced PD mice and the potential neurogenesis function on injured DA neurons, and we also investigated the potential signaling pathways underlying these effects.

## RESULTS

### Cannabinoid CB2R agonist AM1241 attenuates MPTP-induced motor deficits on the Rotarod test

**Figure 1:**
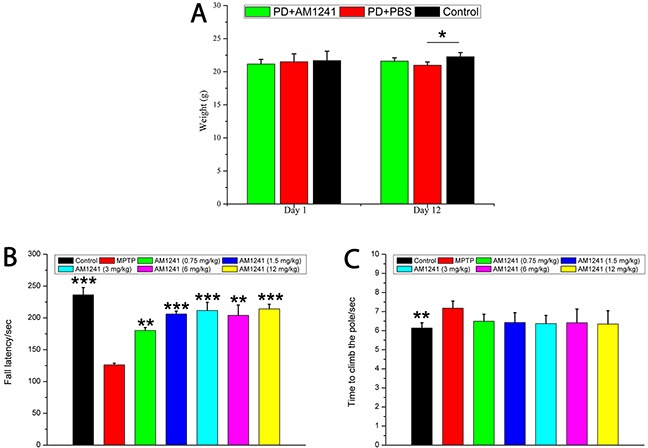
The weight and behavioral characteristics of mice treated with MPTP and AM1241 **(A)** Changes of mice weight in each group; **(B)** fall latency of mice in different groups in the rotarod test, AM1241 reversed the behavioral score of PD mice in a dose dependent manner; **(C)** AM1241 partially protects from the MPTP-induced bradykinesia in the pole test. The values were represented as the means ± S.E.M.; *P<0.05 and ***P<0.001.

As shown in Figure 1(A), PD model mice treated with MPTP had a loss of weight compared to the control group, and this was reversed following treatment with AM1241 in PD mice. As shown in Figure 1(B), an overall difference between AM1241- and PBS-treated PD mice was found in the Rotarod performance (P < 0.001). Mice in the MPTP group had an obvious shorter dropping latency than those of the control group, which confirms that MPTP induced motor coordination deficits. Compared with those of MPTP group, the dropping latency increased significantly with the increase of AM1241 dose. These results demonstrate that AM1241 reversed MPTP-induced motor deficits effectively.

### Cannabinoid CB2R agonist AM1241 attenuates MPTP-induced bradykinesia in the Pole test

Dyskinesia occurs in the majority of patients with PD and MPTP-induced mice models of PD. Therefore, we implemented the Pole test on day 5 after MPTP injection in order to measure bradykinesia. As shown in Figure 1(C), the MPTP group took significantly longer to climb the pole than the control group (p < 0.01). Treatment of the MPTP group with AM1241 reduced climbing times; while this was suggestive of a positive effect of treatment on bradykinesia, this difference was not significant.

### Plasmid concentration levels in the brain of mice treated with AM1241

**Figure 2:**
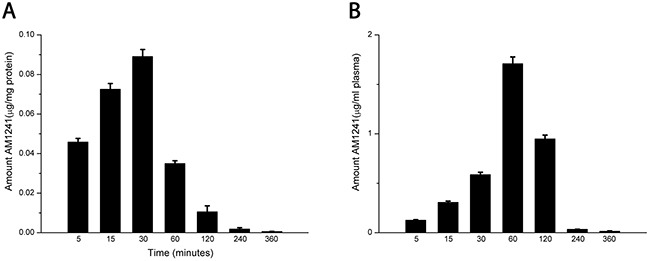
Plasmid concentration and brain distribution of AM1241 in mice The values were represented as the means ± S.E.M.

As shown in Figure 2(A), the concentration of AM1241 in the mice brain reached a maximum at 30 minutes, dropped dramatically to less than half of the maximum at 60 minutes, and to almost zero at 360 minutes. As shown in Figure 2(B), the maximum plasmid concentration of AM1241 in mice was seen at 60 minutes and dropped to almost zero at 240 minutes. This limited retention time of AM1241 *in vivo* demonstrated that AM1241 is metabolized quickly and the pharmacokinetics of AM1241 conformed with one compartment open model.

### Cannabinoid CB2R agonist AM1241 increases dopamine and serotonin levels in PD mice

**Figure 3:**
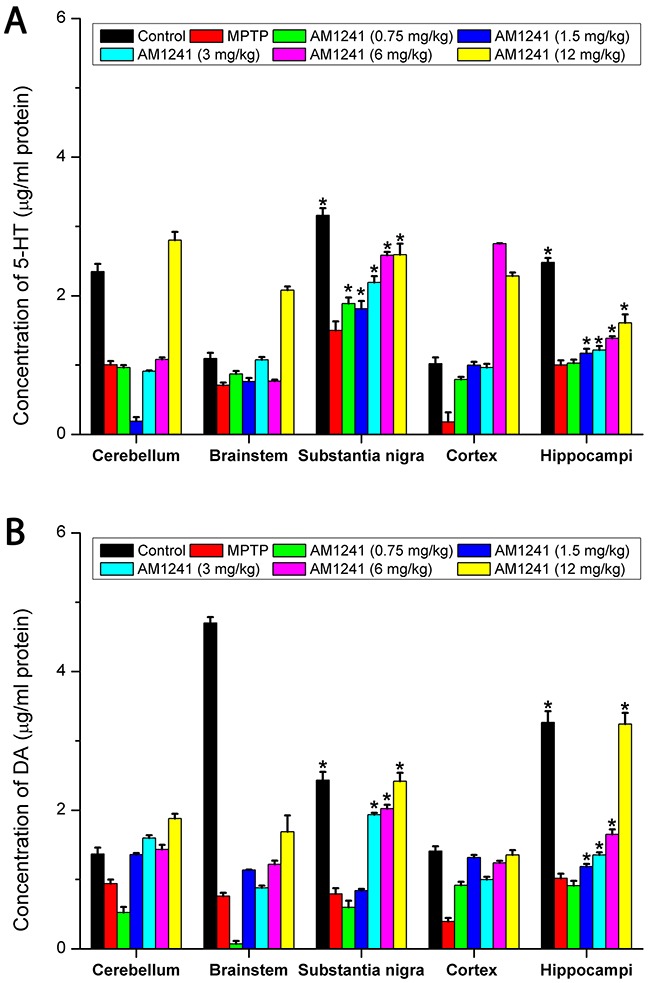
The effect of AM1241 on MPTP-induced striatal DA **(A)** and 5-HT levels **(B)**. MPTP treatment significantly decreased DA and 5-HT levels compared to control, but AM1241 treatment prevented MPTP-induced decrease in DA and 5-HT levels. The values were represented as the means ± S.E.M.

Using high-performance liquid chromatography (HPLC), we quantified the striatal DA and serotonin (5-HT) levels. As shown in Figure 3(A), there was a significant decrease in 5-HT levels following MPTP-treatment. 5-HT levels could be significantly increased with increasing of the concentrations of AM1241. As shown in Figure 3(B), the results revealed a significant decrease (32.6%) of DA levels in the MPTP-treated mice compared to the PBS-treated mice (P < 0.05). However, DA levels significantly increased in a dose dependent manner following administration of AM1241, and recovered up to the level of control group when the dose of AM1241 was 12 mg/kg. This recovery function of AM1241 on DA and 5-HT levels indicated that AM1241 could effectively increase the levels of monoamine neurotransmitter in PD mice.

### AM1241 effects CB2 expression and reverses PD through regulation of Parkin/PINK1 and PI3K/AKT/MEK in the substantia nigra and hippocampus

**Figure 4:**
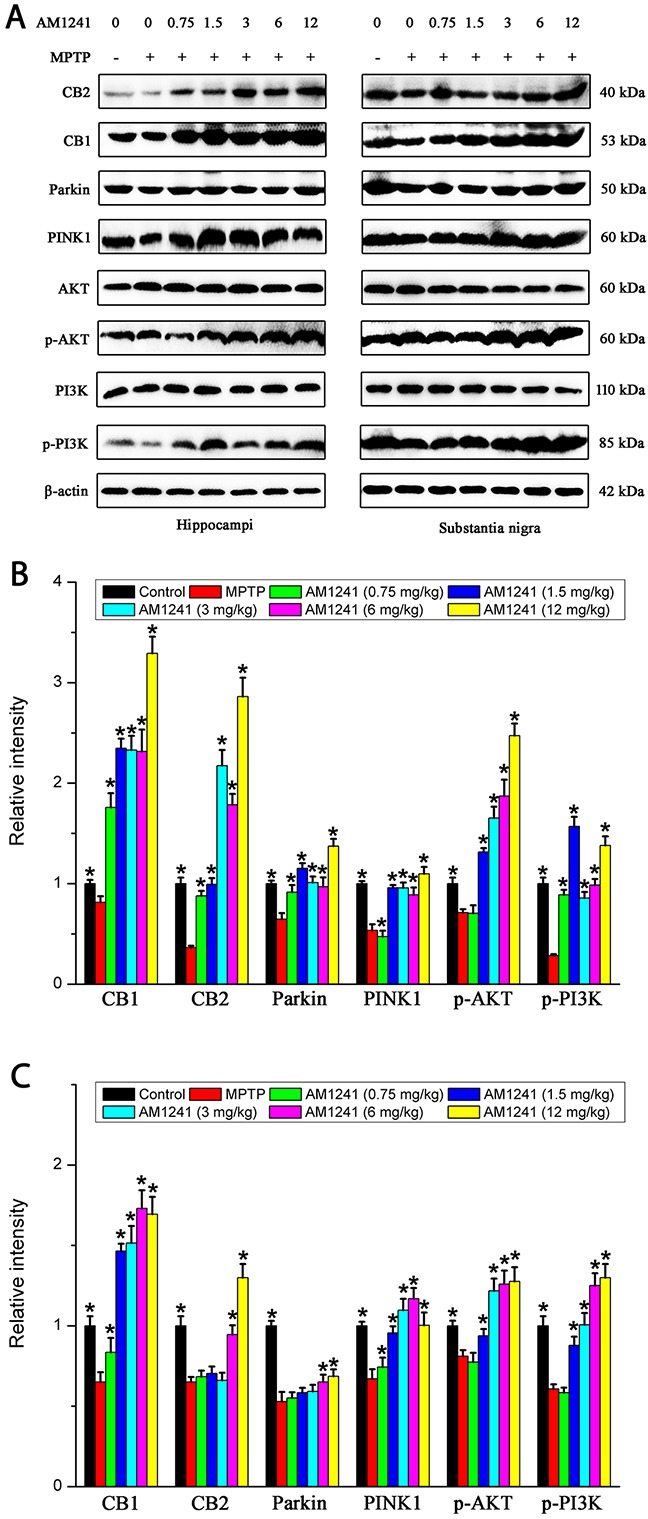
Levels of CB1, CB2, PINK1, Parkin, p-PI3K and p-AKT in the substantianigraand hippo of mice of control group, MPTP group and AM1241 treatment groups **(A)** Representative Western blot bands of these proteins in the substantianigra and hippo of mice. **(B)** Quatified level of these proteins. The values were represented as the means ± S.E.M.

Inactivation of Parkin/PINK1 and PI3K/AKT/MEK results in mitochondrial dysfunction and increased sensitivity to oxidative stress. Thus, Parkin, PINK1, PI3K, AKT, and MEK expressions likely protect against oxidative stress in cells via a common mechanism. The expressions of CB1, CB2, Parkin, PINK1, p-PI3K, and p-AKT were determined by both western blot assay and immunostaining. As shown in Figure 4(A), 4(B), and 4(C), both the western blot bands and quantify analysis revealed a significant decrease in the expression of CB1, CB2, Parkin, PINK1, p-PI3K, and p-AKT of PD mice compared to untreated control, both in the substantia nigra and hippocampus. Interestingly, the decrease in the level of these proteins could be effectively reversed by the treatment of AM1241, in a dose dependent manner. Namely, AM1241 significantly increased CB2R expression and up-regulated Parkin/PINK1 and PI3K/AKT, much higher than that of normal controls, which finally alleviate PD symptoms in MPTP-treated mice.

**Figure 5:**
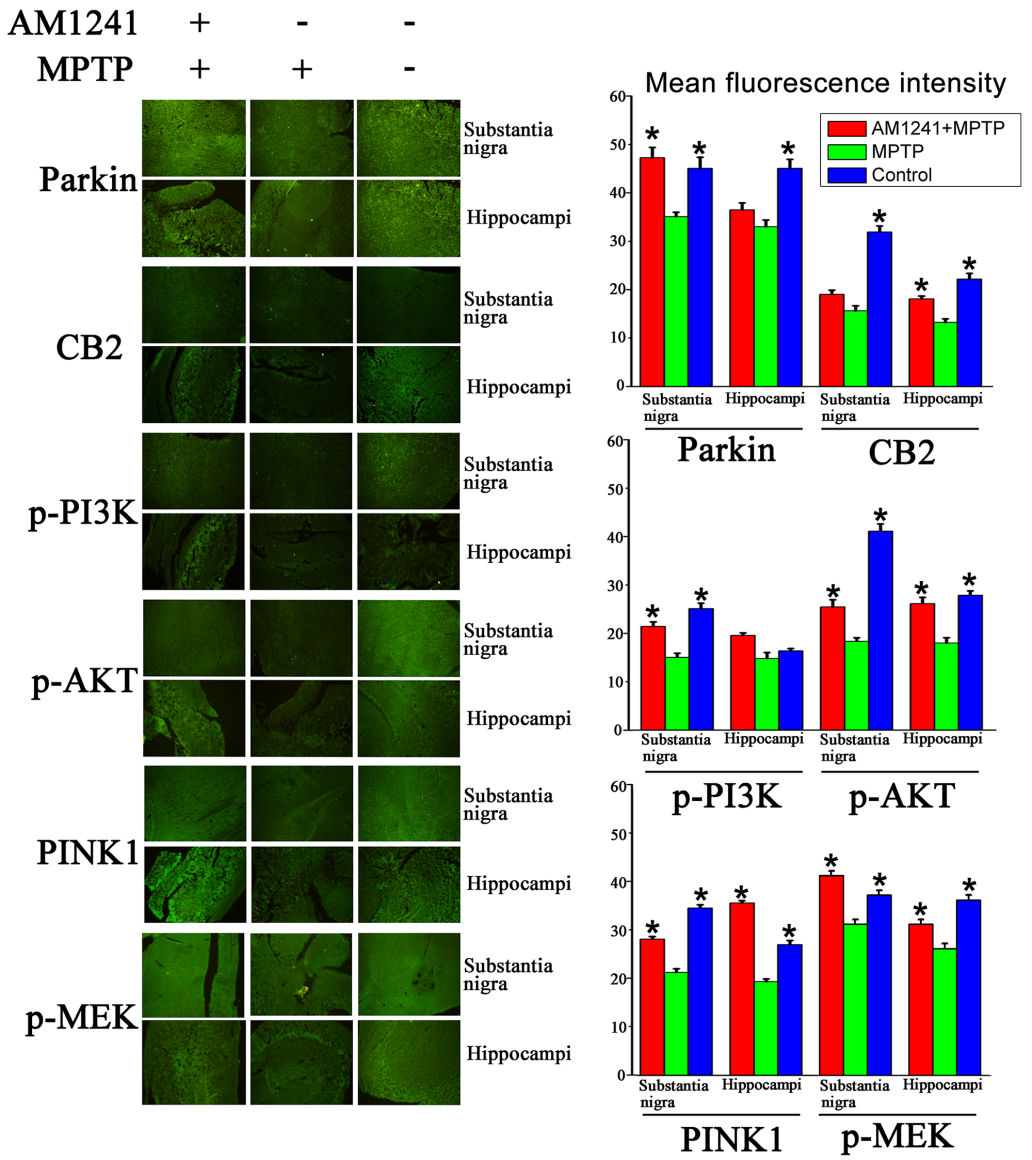
Immuno staining and quantification graphs of Parkin, CB2, p-AKT, p-PI3k, PINK1 and MEK in substantianigra and hippo of control mice, mice exposed to MPTP, mice exposed to MPTP and treated with AM1241 AM1241 treatment restored the decrease of expressions of these proteins in substantianigra and hippo of mice exposed to MPTP. The values were represented as the means ± S.E.M.

It was also clearly evident from immunofluorescence analysis that MPTP exposure decreased Parkin, CB2, p-AKT, p-PI3K, PINK1, and MEK expressions in the substantia nigra and hippocampus (Figure 5). Furthermore, PD mice treated withAM1241 resulted in an up-regulation of Parkin, PINK1, p-PI3K, p-AKT, and MEK expression in substantia nigra and hippocampus compared to PD mice with no AM1241 treatment. These results suggested that AM1241 was capable of attenuating MPTP intoxication via the up-regulation of CB2R expression and activating PI3K/AKT signaling pathways in the substantia nigra and hippocampus of MPTP-induced PD mice.

### AM1241 increases mRNA expression of CB1, CB2, Parkin, and PINK1 of PD mice

**Figure 6:**
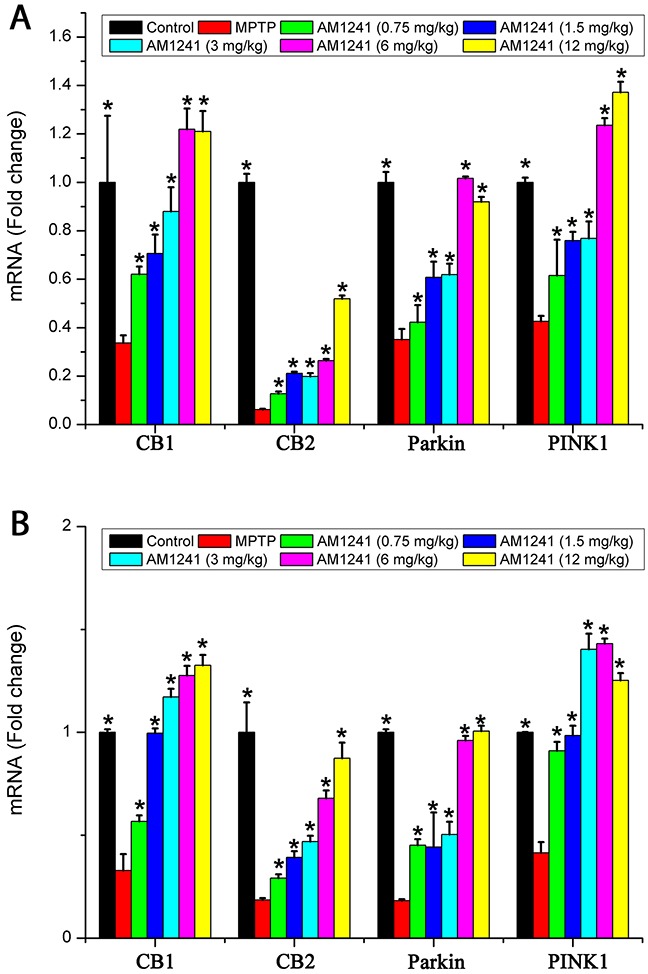
mRNA levels of CB1, CB2, Parkin and PINK1 in the substantianigra of control mice, MPTP group and AM1241 treatment groups The values were represented as the means ± S.E.M.

The mRNA levels of CB1, CB2, Parkin, and PINK1 were obtained by RT-PCR to further explore the mechanism of AM1241 on PD mice. It could been clearly seen that, compared to control group, the mRNA levels of CB1, CB2, Parkin, and PINK1 in the substantia nigra (Figure 6(A)) and hippocampus (Figure 6(B)) were sharply reduced in PD mice following AM1241 treatment. After the treatment of AM1241, CB1, CB2, Parkin, and PINK1 mRNA levels were highly induced, of which the tendency was in accordance with that of protein level. The results of mRNA expressions further demonstrated that AM1241 treated PD by activating CB2R and Parkin/PINK1 signaling pathway.

### Neurogenesis in the substantia nigra of AM1241 treated PD mice

**Figure 7:**
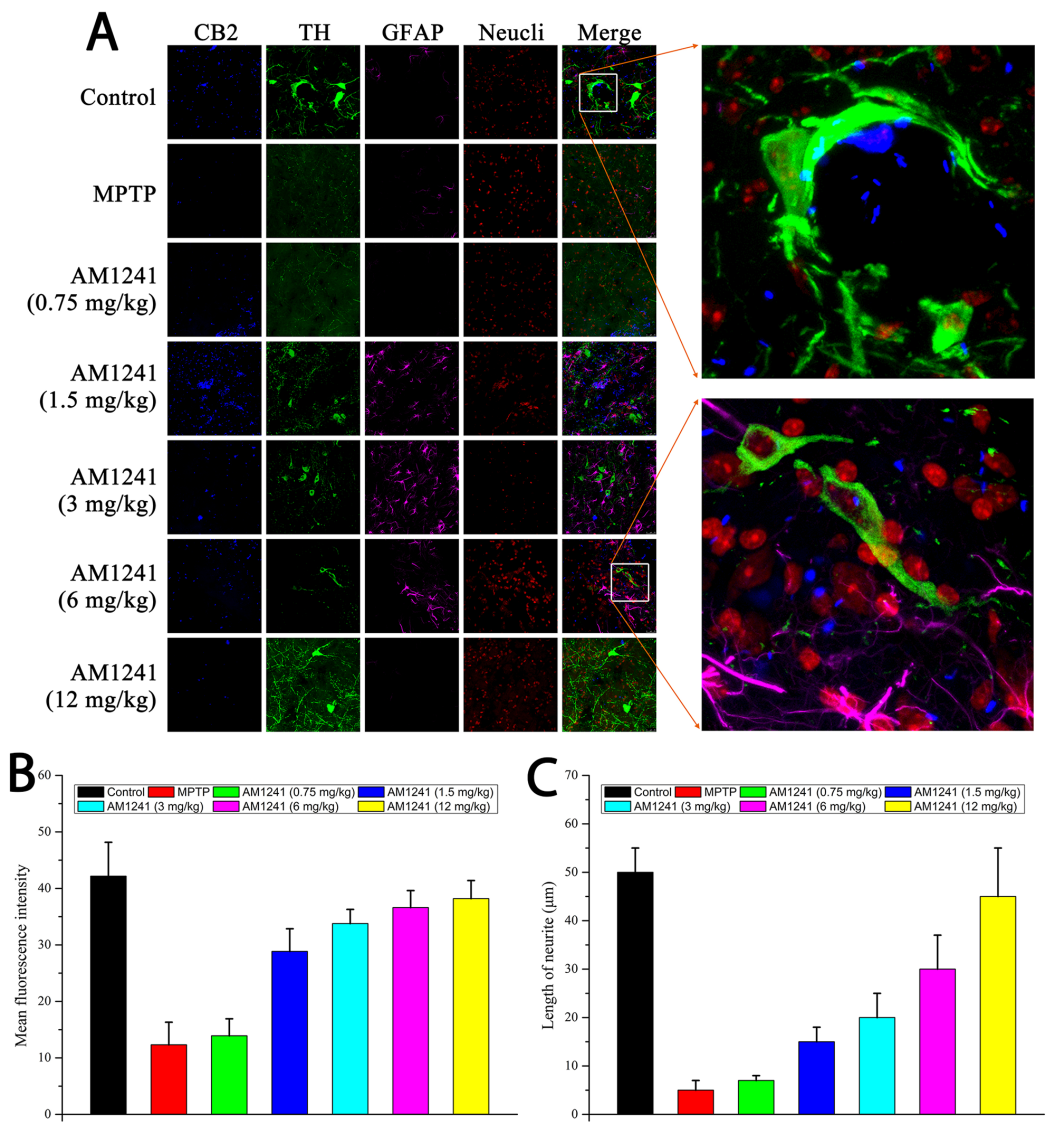
Immuno staining for neurogenesis in substantia nigra of MPTP-induced PD mice **(A)** TH-positive cells represent for DA neurons, GFAP-positive cells represent for astrocytes. The enlarge graphs show the clear axons of DA neurons and the co-localizations of CB2 receptor and TH-positive cells. **(B)** Histogram of quantification of TH positive cells by mean fluorescence intensity. **(C)** Histogram of quantification of length of neurite.

**Figure 8:**
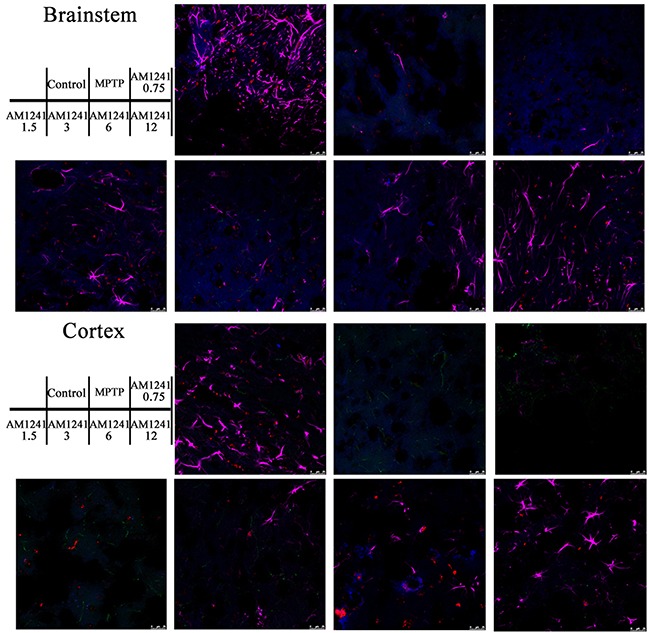
Immunostaining of microglia in substantianigra of MPTP-induced PD mice IBA1-positive cells represent for microglia, only a few co-localizations of CB2 receptor and IBA1-positive cells could be seen.

To examine the effects of AM1241 on DA neuronal regeneration, we employed the known marker, tyro-sine hydrolyse (TH), for DA neurons, and used the GFAP marker for astrocytes in the substantia nigra, as shown in Figure 7. Compared to the control group, MPTP induced significant cell death of DA neurons and few TH-positive cells were detected in the brains of mice. However, more and more DA neurons were observed with increasing concentrations of AM1241, especially when the concentration was higher than 1.5 mg/kg. When the concentration of AM1241 was as high as 6 mg/kg, TH-marked DA neurons, as well as axons, could be clearly observed. Furthermore, the co-localization of CB2R and TH-positive cells could been seen from both the control sample and the AM1241 (6 mg/kg) treated PD brain sample, which further demonstrated the function of AM1241 on PD mice via activation of the CB2R and regeneration of DA neurons. In addition to this, AM1241 treatment increased the number of GFAP-positive astrocytes, which might also play a role in neurogenesis and PD treatment. As shown in Figure 8, we used IBA1 as the marker of microglia; the co-localization of CB2R and IBA1-positive cells was far fewer, which might indicate that microglia participated less in the neurogenesis function of AM1241.

**Figure 9:**
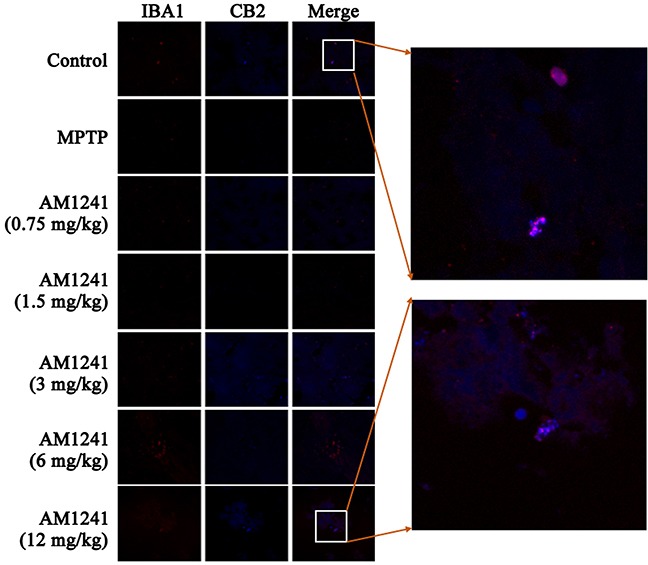
Immunostaining for neurogenesis in cortex and brainstem of MPTP-induced PD mice Nearly no TH-positive cells were observed. The regeneration of GFAP -positive astrocytes was enhanced with the increase of the dose of AM1241.

As shown in Figure 9, we observed few TH-positive cells in regions of the cortex and brainstem, in all samples. Compared to control group, nearly no GFAP-positive cells remained in MPTP treated mice. However, with the increase in the dose of AM1241, the amount of astrocytes reversed to the level of the control group, both in the cortex and brainstem, which indicated that AM1241 inspired the regeneration of astrocytes. The neurogenesis function of AM1241 on DA neurons and astrocytes suggested that AM1241 could be an effective drug for neuronal injury recovery, such as that seen in PD.

## DISCUSSION

MPTP is a commonly used chemical to induce a PD-like state in rodents [13–14]. As animals do not develop PD, the MPTP lesion model is one of several models that are used to investigate the underlying mechanisms of PD and to test novel compounds for their neuroprotective properties [15]. This study investigated the neurogenesis and PD recovery effects of CB2R agonist, AM1241, treatment in MPTP-induced PD mice. We found that AM1241 significantly inhibited MPTP-mediated neural toxicity. Our *in vivo* data showed that administration of AM1241 significantly enhanced neurogenesis of DA neurons in the substantia nigra, in a dose dependent manner. Furthermore, western blot assay and immunostaining results revealed that the PI3K/AKT signaling pathway was involved in the action of AM1241.

Our results consistently demonstrated that the cannabinoid CB2R agonist AM1241 showed strong protection from the impairments induced by MPTP treatment. Previous findings have shown that CB2R expression increases following a neuroinflammatory event [16] and that cannabinoid agonists can modulate microglia mobility via activation of CB2Rs both *in vitro* [17] and *in vivo* [18]. Others have reported that CB2R is up-regulated following 3-day MPTP treatment, and the protective effect was come from the inhibition of microglial activation [8]. However, García et al. found a significant down-regulation of CB2R expression in patient with PD [6]. In addition, in 6-OHDA-induced PD rats, CB2R was found a process of down-up-down regulation [19]. Together, these findings confirm the regulatory function of CB2 in PD; similarly, in our study, MPTP-induced PD mice showed a down-regulation of CB2Rs three weeks after MPTP injection. These findings suggest that CB2Rs are critical for protection against MPTP toxicity. Furthermore, stimulation of CB2Rs reduces the neuroinflammatory process that occurs in animal models of amyotrophic lateral sclerosis and multiple sclerosis [20–21].

One key question in the treatment of PD is how to effectively recover the massive loss of DA neurons in the substantia nigra. A specific agonist of the CB2R, AM1241, has typically been reported as a candidate for pain [11] and stroke [10] treatment; the pre-treatment but not post-treatment protective function in stroke has been confirmed in rats. Only one study reported that AM1241 enhanced neurogenesis in GFAP/GP120 transgenic mice [12]. Thus, until now, the treatment function and neurogenesis effect of AM1241 on PD has remained unexplored. We firstly proved the good biocompatibility of AM1241 (the maximum dose used in the treatment) on normal mice as shown in Supplementary Figure 1, in addition, AM1241 did not affect the level of DA and 5-HT on normal mice under the experimental concentration as shown in Supplementary Figure 2. Secondly, our results demonstrated the post-treatment protective function and neurogenesis effect of AM1241 on MPTP-induced PD mice *in vivo*. The PD mice displayed severe DA neuron damage in the substantia nigra; however, after administration of AM1241, significant *in vivo* neurogenesis was observed, as indicated by a significant increase in the number of TH-positive cells.

We found that the neuroprotective effect of AM1241 was mediated via an enhanced expression of Parkin/PINK1. Meanwhile, MPTP reduced the activation of p-AKT and p-PI3K, and AM1241 treatment reversed this to some extent. Consistent with these results, it was reported that the activation of PI3K/AKT and ERK signaling pathway was involved in the protection of DA neurons and attenuation of neuroinflammation [22]. Neuroinflammation is a vital problem of PD that induces progressive DA neuron loss [23]. Related research also found that by using SH-SY5Y cells, MANF protected cells against 6-OHDA-induced toxicity via activation of the PI3K/Akt/mTOR pathway [24]. Considering previous work and the present experimental results together, one possible mechanism underlying the neurogenesis effect of AM1241 on DA neurons might be the modulation of the PI3K/AKT signaling pathway.

Based on the results presented in this study, we propose that the selective CB2R agonist, AM1241, has a significant therapeutic effect on PD and can regenerate DA neurons after the neurotoxic effect of MPTP treatment. These results suggest that AM1241 represents a new candidate for the prevention of the neurodegenerative process, and may even have the potential to cure PD.

## MATERIALS AND METHODS

### Materials

MPTP was purchased from Sigma (St. Louis, MO). AM1241 were obtained from Selleck.cn (Shanghai, China). Dimethyl sulfoxide (DMSO) and Triton X-100 were obtained from Fisher Scientific (Pittsburgh, PA). All the antibodies were from Abcam (Cambridge, MA) or Cell Signaling Technology (Danvers, MA). All other chemicals, unless otherwise stated, were from Sigma (St. Louis, MO).

### Animals and drug treatments

All experiments were carried out in 9–12 week-old, male C57BL/6N mice. Animals were housed at 22±1°C (12-hour light-dark cycle) with *ad libitum* access to food and water for one week before experiments. All experiments were carried out in accordance with the National Institutes of Health Guide for the Care and Use of Laboratory Animals and approved by the Institutional Animal Care and Use Committee of Tongi University.

PD mice: Animals received intraperitoneal (i.p.) injections of either PBS or MPTP (30 mg/kg, i.p.), following previously published guidelines [25]. After 5 days of injections, behavioral tests began.

For specific activation of the CB2R, PD mice were divided into two groups (8 mice per group). Groups received either vehicle (PBS) or cannabinoid drugs (AM1241, from 0.75 to 12 mg/kg), which were administered chronically (i.p.) once a day for 12 days starting 24 h after the last injection of MPTP.

### Behavioral tests

To assess the impact of the unilateral lesions on motor function, the rats were assessed for motor dysfunction using the Rotarod test and the Pole test. In the Rotarod test, mice were evaluated for their motor activity over 300 seconds on a Rotarod at an accelerating speed (from 4 rpm to 40 rpm) [26, 27]. The time they could spend on the revolving rod was measured. All mice were pre-trained on the Rotarod apparatus in order to reach a stable behavioral performance, whereby mice completed 3 training sessions each and the average retention time on the revolving rod was recorded. In the Pole test, mice began at the top of a pole (diameter, 0.8 cm; height 60 cm) and the time taken to climb down the pole was recorded, according to the protocol previously described [28, 29]. This motor performance was measured 3 times and the average time calculated.

### Blood and brain distribution of AM1241

Male, 9–12-week-old C57BL/6N mice were treated with AM1241 (12 mg/kg), mice were euthanized at different time points, and the plasmid and brain were collected and separated. Plasmid of 200 μl was mixed adequately with 400 μl of acetonitrile and centrifuged at 10,000 RPM for 15 min at 4°C. The supernatant was filtered through a nylon syringe filter (0.2 μm) and samples (20 μl) were injected into a 150 ± 2 mm ODS C18 column. The plasmid concentrations of AM1241 were assayed by HPLC detection. Brains of treated mice were homogenized in 300 μl PBS and mixed with 600 μl acetonitrile, vortex, and centrifuged at 10,000 RPM for 15 min at 4°C to obtain the supernatant to inject into the C18 column for the following HPLC detection. The protein levels of brain samples were detected using the BCA Protein Assay Kit. Levels of AM1241 in the mice brains at different time points were equaled by protein level.

### Immunohistochemistry

The mice brains were kept in 4% ice-cold paraformaldehyde for fixing for 4 h and cryoprotected in 20% sucrose/PBS for at least 48 h. Then, they were imbed in optimal cutting temperature compound and sliced into 7-μm sections. For double-immunofluorescence staining, the sections were incubated in primary antibodies (1:100 dilution) overnight at 4°C. The following primary antibodies or dyes were used: Anti-Cannabinoid Receptor II antibody (Abcam, MA, US), anti-Parkin (Cell Signaling Technology, Inc), anti-PINK1 (Cell Signaling Technology, Inc), anti-pPI3K (Cell Signaling Technology, Inc), anti-pAKT (Cell Signaling Technology, Inc.), and anti-MEK (Cell Signaling Technology, Inc). After washing in PBS, sections were treated simultaneously with a mixture of FITC-conjugated rabbit anti-rat IgG (1:200 dilution; Vector Laboratories) for 1 h at room temperature. For one-step immunofluorescence staining, the following dyes conjugated antibodies were used: Rabbit polyclonal Anti-Cannabinoid Receptor II (ab3560) conjugated with CF405S (Biotium), rabbit monoclonal Anti-Tyrosine Hydroxylase (Alexa Fluor® 488) (ab192463) for dopaminergic neurons, mouse monoclonal Anti-GFAP (Cy3 ®) (ab49874) for astrocytes, rabbit monoclonal anti-IBA1 (Alexa Fluor® 647) (ab195032) for microglia, and RedDot1 (Biotium) as a nuclei marker. Slices were observed under a confocal laser scanning microscope (Leica TCS SP5 II). The quantitation of DA neurons was determined by mean fluorescence intensities of TH expression and length of neurites using Image-Pro Plus 6.0.

### Western blotting

The mice brains were homogenized in lysis buffer (50 mM Tris-HCl, 150 mM NaCl, 1 mM Na3VO4, 10 mM NaF, 1 mM EDTA, 0.1% Triton X-100, 0.1% SDS) containing protease inhibitors. Equal amounts of protein were separated by SDS-polyacrylamide gel (7.5% or 10% for MAC-1 immunoblotting) and transferred to nitrocellulose membranes (0.45 μm, Millipore; Billerica, MA). Non-specific binding was blocked with TBS-T (50 mM Tris-HCl pH 7.6, 150 mM NaCl, and 0.1% Tween-20) containing 5% non-fat milk for 1 hr at room temperature. The membranes were then incubated overnight at 4°C with one of the following primary antibodies: anti-CB1, anti-CB2, anti-Parkin, anti-PINK1, anti-pPI3K, anti-pAKT, or anti-β-actin. After 3 washes in TBS-T, membranes were incubated with goat anti-mouse, anti-rabbit, or anti-rat HRP for 1 hr at room temperature. The signal was visualized using an ECL chemiluminescence kit (Amersham Biosciences/GE Healthcare; Piscataway, NJ), followed by densitometry using Image J (V.2.1.4.7).

### Real-time PCR

Total RNA was isolated from 0.1 g skin tissue using Trizol reagent (Invitrogen) according to the manufacturer's protocols. The concentration and purity of RNA samples were conducted by Nanodrop ND-2000 (Thermo Science, USA). cDNA was synthesized rimer Script Reverse Transcriptase Kit (Takara). Quantitative real-time PCR was performed using SYBR Premix Ex Taq™ (Takara) on the QuantStudio 7 Flex Real-Time PCR System. The primer sequences (Sangon Biotech, China) are listed in (Table 1). Relative amounts of mRNA were calculated by the relative quantification (ΔΔCt) method. β-actin served as the control gene and the mRNA levels of specific genes were normalized to β-actin.

**Table 1:** Primer sequences for real-time PCR of target genes

Primer name	Primer sequence
**β-actin**	Forward Primer: 5’–AAATCGTGCGTGACATCAAAGAGAA–3’
	Reverse Primer: 5’–ACCCAAGAAGGAAGGCTGGAAAA–3’
**CB1**	Forward Primer: 5’–TCAAGGAGAACGAGGACAACA–3’
	Reverse Primer: 5’–CCAGGGTGAGGGACAGGA–3’
**CB2**	Forward Primer: 5’–ACGGTGGCTTGGAGTTCAAC–3’
	Reverse Primer: 5’–GCCGGGAGGACAGGATAAT–3’
**Parkin**	Forward Primer: 5’–CGTGTGATTTTTGCCGGGAAG–3’
	Reverse Primer: 5’–GGTCCACTCGTGTCAAGCTC–3’
**PINK1**	Forward Primer: 5’–CACACTGTTCCTCGTTATGAAGA–3’
	Reverse Primer: 5’–CTTGAGATCCCGATGGGCAAT–3’

### HPLC with electrochemical detection

Frozen samples from each group were suspended in 300 μl PBS and homogenized for 30 s, mixed with 600 μl of acetonitrile, vortex, and then centrifuged at 10,000 RPM for 15 min at 4°C. The supernatant was filtered through a nylon syringe filter (0.2 μm) and samples (20 μl) were automatically injected using an ESA 542 refrigerated autosampler (ESA, Inc.) onto a 150 ± 2 mm ODS C18 column. The mobile phase, containing 80 mM sodium dihydrogenphosphate monohydrate, 2.0 mM 1-octanesulfonic acid sodium salt, 100 μl triethylamine, 5 nM EDTA, and 10% acetonitrile, pH 3.0, was perfused at 0.25 ml/min. Under these conditions, the concentrations of dopamine (DA) and serotonin (5-HT) were measured.

### Data analysis

The statistical significance of the differences between the groups was determined using Student's t-test, and one-way analysis of variance (ANOVA) was used for the experiments with multiple groups. For all statistical analyzes, a P < 0.05 was considered significant. All data are expressed as mean±SEM.

## SUPPLEMENTARY FIGURES



## Abbreviations

PD: Parkinson's disease
DA: dopaminergic/dopamine
SNc: substantianigra compact
L-dopa: levodopa
CB1R and CB2R: the type 1 and type 2 cannabinoid receptors
CB2: type 2 cannabinoid
DMSO: dimethyl sulfoxide
i.p.: intraperitoneal
PBS: phosphate buffer saline
MPTP: 1-methyl-4-phenyl-1,2,3,6-tetrahydropyridine
RPM: r/min
HPLC: High Performance Liquid Chromatography
FITC: fluorescein isothiocyanate
RT: room temperature
ODS: Octadecylsilyl
EDTA: Ethylenediaminetetraacetic acid
5-HT: 5-hydroxytryptamine
6-OHDA: 6-hydroxydopamine
TH: Tyrosine hydroxylase
