# Supplementary material for: AM1241 alleviates MPTP-induced Parkinson's disease and promotes the regeneration of DA neurons in PD mice
Source: Oncotarget. 2017 Jun 29;8(40):67837–50. doi: 10.18632/oncotarget.18871 (PMC5620217; doi:10.18632/oncotarget.18871)
Supplement: Supplementary file 1 [file oncotarget-08-67837-s001.pdf]

# AM1241 alleviates MPTP-Induced Parkinson's Disease and promotes the regeneration of DA neurons in PD mice

## SUPPLEMENTARY FIGURES

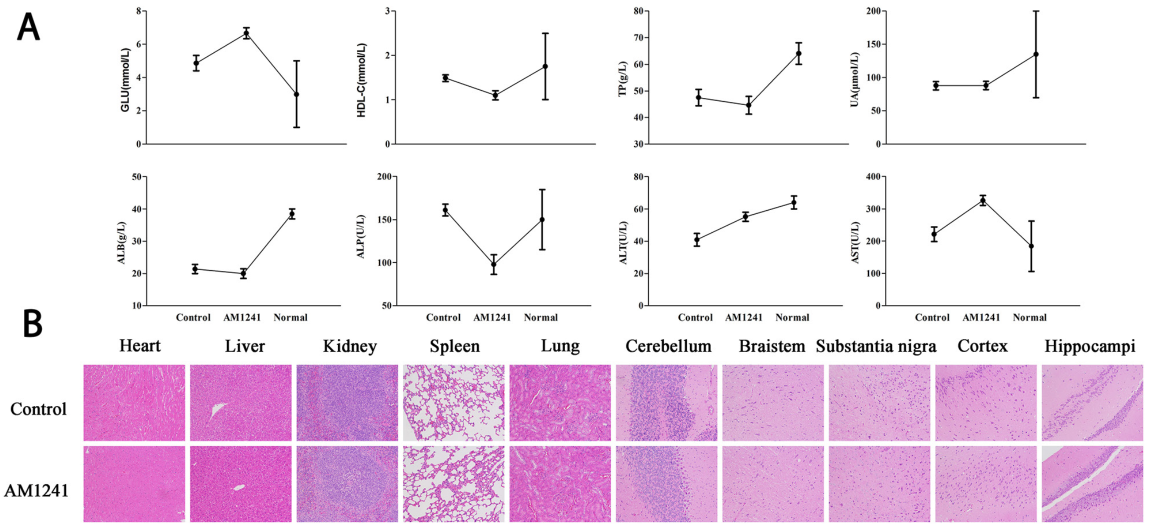


**Supplementary Figure 1: Biocompatibility of AM1241 on normal mice. Control mice was treated with AM1241 (12 mg/kg, the maximum dose in our experiment) for 2 weeks before detections. (A)** blood biochemical analysis of mice treated with AM1241 of 12 mg/kg. **(B)** HE staining of mice organs treated with AM1241, which revealed that AM1241 did not show significant toxicity on each organs.

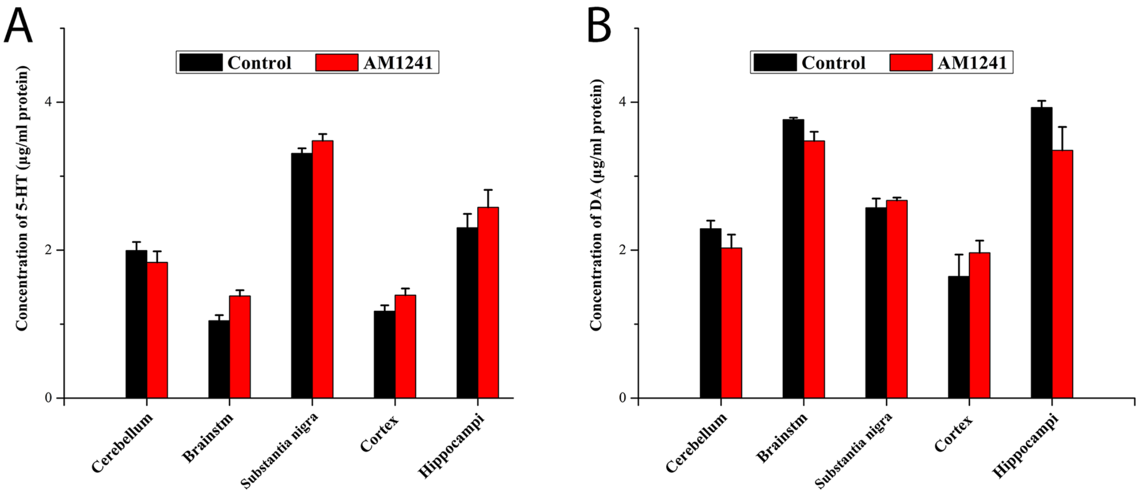


**Supplementary Figure 2: The detections of concentrations of DA and 5-HT in different regions of mice brain that treated only with AM1241 of 12 mg/kg.** The results confirmed that AM1241 did not affect the level of DA and 5-HT on normal mice under the experimental concentration.

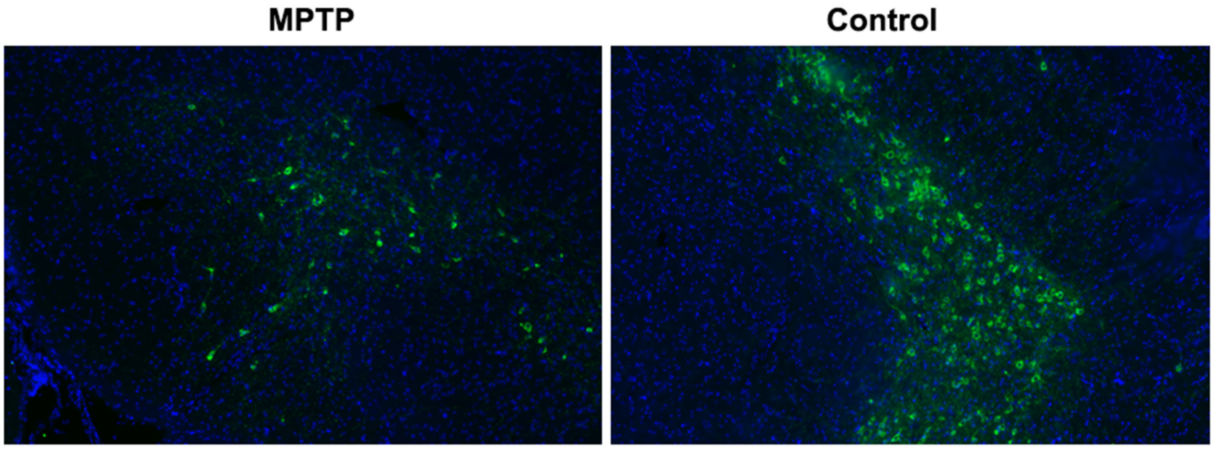


**Supplementary Figure 3: The immunofluorescence antibody assay of TH positive cells in the striatum of control mice and MPTP model.**
